# Modulation of Systemic Immune Responses through Commensal Gastrointestinal Microbiota

**DOI:** 10.1371/journal.pone.0053969

**Published:** 2013-01-11

**Authors:** Kyle M. Schachtschneider, Carl J. Yeoman, Richard E. Isaacson, Bryan A. White, Lawrence B. Schook, Maria Pieters

**Affiliations:** 1 Department of Animal Sciences, University of Illinois, Urbana, Illinois, United States of America; 2 Institute for Genomic Biology, University of Illinois, Urbana, Illinois, United States of America; 3 Department of Animal and Range Sciences, Montana State University, Montana, United States of America; 4 Department of Veterinary and Biomedical Sciences, University of Minnesota, St. Paul, Minnesota, United States of America; Baylor College of Medicine, United States of America

## Abstract

Colonization of the gastrointestinal (GI) tract is initiated during birth and continually seeded from the individual’s environment. Gastrointestinal microorganisms play a central role in developing and modulating host immune responses and have been the subject of investigation over the last decades. Animal studies have demonstrated the impact of GI tract microbiota on local gastrointestinal immune responses; however, the full spectrum of action of early gastrointestinal tract stimulation and subsequent modulation of systemic immune responses is poorly understood. This study explored the utility of an oral microbial inoculum as a therapeutic tool to affect porcine systemic immune responses. For this study a litter of 12 pigs was split into two groups. One group of pigs was inoculated with a non-pathogenic oral inoculum (modulated), while another group (control) was not. DNA extracted from nasal swabs and fecal samples collected throughout the study was sequenced to determine the effects of the oral inoculation on GI and respiratory microbial communities. The effects of GI microbial modulation on systemic immune responses were evaluated by experimentally infecting with the pathogen *Mycoplasma hyopneumoniae*. Coughing levels, pathology, toll-like receptors 2 and 6, and cytokine production were measured throughout the study. Sequencing results show a successful modulation of the GI and respiratory microbiomes through oral inoculation. Delayed type hypersensitivity responses were stronger (p = 0.07), and the average coughing levels and respiratory TNF-α variance were significantly lower in the modulated group (p<0.0001 and p = 0.0153, respectively). The *M. hyopneumoniae* infection study showed beneficial effects of the oral inoculum on systemic immune responses including antibody production, severity of infection and cytokine levels. These results suggest that an oral microbial inoculation can be used to modulate microbial communities, as well as have a beneficial effect on systemic immune responses as demonstrated with *M. hyopneumoniae* infection.

## Introduction

The mammalian gastrointestinal (GI) tract is home to a complex microbial community with a population over 10 times greater than the total number of somatic cells present in the host [1]. Early life environmental stimuli are important in establishing the GI microbiota as well as developing the host immune system. Colonization of the GI tract starts at birth with exposure to bacteria from the mother and the surrounding environment. Germ-free animal studies have shown that GI microbiota and their hosts do not simply co-exist, but rather form a mutualistic relationship [1]. Some benefits accounted for by this relationship include sharing of nutrients and organic substrates, pathogen colonization resistance, regulation of fat storage and maturation and modulation of gastrointestinal immunity [1], [2]. The composition of an individual’s GI microbiota is dependent on a number of factors, including early environmental exposures, hygiene and diet [3], [4], [5].

The central role of GI microorganisms in developing and modulating host intestinal immune responses has been a subject of investigation over the last few decades [6]. Animal studies using pigs raised in indoor or outdoor environments have demonstrated differences in mucosa-adherent microbial diversity as well as increased gastrointestinal immune gene expression levels in indoor-housed pigs [7], while another study has shown that the time and length of exposure to microbes early in life may be crucial in establishing the porcine GI microbiota [8]. There is also increasing evidence of strong associations between particular GI microbial populations and the incidence of enteric and/or metabolic disorders, such as obesity and diabetes [9], [10], as well as differential intestinal immune responses [7]. In addition, recent studies have shown the successful use of GI microbial modulation as a therapy to combat chronic *Clostridium difficile* infections and other GI conditions in humans [11], [12], [13].

The GI microbiota are in constant contact with the epithelial surfaces of the intestinal mucosa, where they interact with dendritic cells (DC) in Peyer’s patches [14]. The microbe-associated molecular patterns present in the gut microbiota are recognized by various DC pattern recognition receptors, such as toll-like receptors (TLRs), which migrate into mesenteric lymph nodes, where the antigens are bound to MHC class II receptors and presented to T cells, causing activation and differentiation [14]. This process serves as a bridge between GI microbiota and the systemic immune system, and helps to explain how GI microbial diversity is involved in the development and regulation of immune responses outside of the GI tract. This interaction, as well as the hygiene hypothesis, which proposes that infections in early childhood and unhygienic contact with older siblings and the environment mitigate allergic diseases [15,16], has led to the testable hypotheses that GI microbiota could modify the hosts immune responses outside the GI tract [17], [18]. However, the full spectrum of early GI tract stimulation and the subsequent modulation of systemic immune responses are far from understood, and even more uncertain is how the GI microbiota may be modulated and subsequently serve as a therapeutic tool.

The swine respiratory pathogen *Mycoplasma hyopneumoniae* (*M. hyopneumoniae)* was chosen as the pathogenic challenge for this study. *M. hyopneumoniae* infection is tissue specific and results in a chronic respiratory disease characterized by coughing, lung lesions, and decreases in daily gain, as well as predisposes animals to other respiratory diseases of bacterial and viral origin [19], [20]. The microscopic hallmark of swine mycoplasmosis is a strong immune response, evident by perivascular and peribronchial lymphoproliferation [20] that ultimately accounts for lung consolidation, resulting in pneumonia. Humoral and cellular immune responses following infection and/or vaccination [21] have been demonstrated in pigs of all ages.

**Figure 1:**
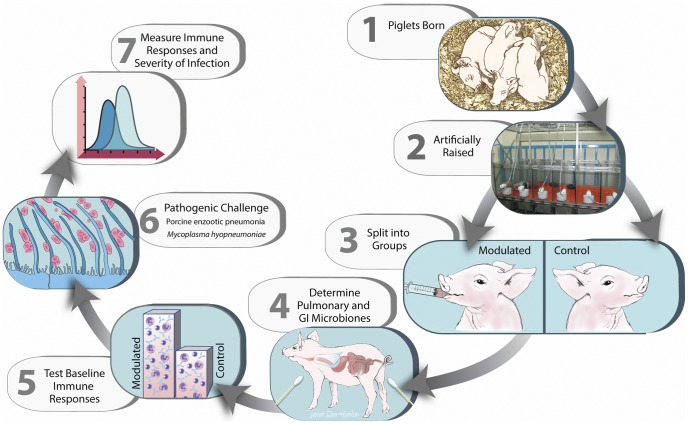
Experimental timeline. 1. A litter of pigs (12) was removed from their mother immediately following birth in order to prevent exposure to the maternal GI microbiota. 2. The pigs were raised in controlled research units and fed medicated milk replacer until weaning (28 days old). 3. At 33 days of age the pigs were randomly assigned to 2 groups based on weight and gender, one of which was inoculated (modulated) with the GI microbiota from a healthy adult boar for seven consecutive days, while the other was not (control). 4. Nasal swabs and fecal samples were collected throughout the study and sequenced to determine the effects of the oral inoculation on GI and respiratory microbial communities. 5. Allergic and delayed type hypersensitivity responses (type I and IV, respectively) were measured in both groups via *A. suum* worm extract skin testing at 54 days of age. 6. Experimental infection with *M. hyopneumoniae* was performed at 69 days of age. 7. Various systemic immune responses and severities of infection were analyzed throughout the study: *M. hyopneumoniae* antibody production, respiratory TLR2 & TLR6 transcription levels, cytokine and C-reactive protein levels, daily weight gain, bacterial load, coughing and lung lesion scores.

This study demonstrates the use of GI microbial modulation as a therapeutic tool to alter porcine systemic immune responses to a pathogenic challenge by *M. hyopneumoniae*. Briefly, a litter of pigs was removed from their mother immediately following birth and raised in controlled research units until weaning (28 days old), after which the pigs were randomly assigned to 2 groups. One group was inoculated (modulated) with the GI microbiota from a healthy adult boar for seven consecutive days, while the other was not (control). The effects of the oral inoculation on GI and respiratory microbial communities, porcine systemic immune responses and severity of infection following experimental infection with *M. hyopneumoniae* were determined (Figure 1).

## Results

### Gastrointestinal and Respiratory Microbiome Modulation

#### Microbial Diversity

Shannon’s diversity index and Chao1 estimates were used to calculate diversity and richness of the microbial community samples, respectively (Table 1). Before the oral inoculation was performed the average Shannon index was 2.14 and 2.32 for the GI microbiome (feces) of the modulated and control group, respectively. After the oral inoculation, Chao1 estimates suggested a total increase in richness of 656 Operational Taxonomic Units (OTUs) in the modulated GI samples and Shannon’s index also revealed an increase in diversity (3.14 and 2.19, *p* = 0.012; Table 1) compared to the control group, respectively. No significant difference was seen in the respiratory samples for the modulated and control group following oral inoculation, with Chao1 estimates suggesting no increased richness in the modulated group and Shannon’s indices also revealing no increase in diversity compared to the control group for both upper (*p* = 0.7735) and lower respiratory samples (*p* = 0.4555; Table 1). The oral inoculum samples had an average Shannon’s diversity index of 3.56.

**Table 1:** Shannon’s diversity index and chao1 estimates for GI and respiratory microbiome samples.

	Control GI	Modulated GI	Control Upper Respiratory	Modulated Upper Respiratory	Control Lower Respiratory	Modulated Lower Respiratory
	Shannon's Diversity Index		Shannon's Diversity Index		Shannon's Diversity Index	
**Before Inoculation**	2.319810978	2.140615909	NA	NA	NA	NA
**After Inoculation**	2.90892015919303*	3.14141065945317*	3.127766308	3.146193617	1.47	1.49
	**Chao1 Estimate**		**Chao1 Estimate**		**Chao1 Estimate**	
**Before Inoculation**	1545	1389.6	NA	NA	NA	NA
**After Inoculation**	1635.6	2291.7	2541.3	2309.6	394.4	339.7

### OTU Analysis of the Gastrointestinal Microbiome Samples

**Figure 2:**
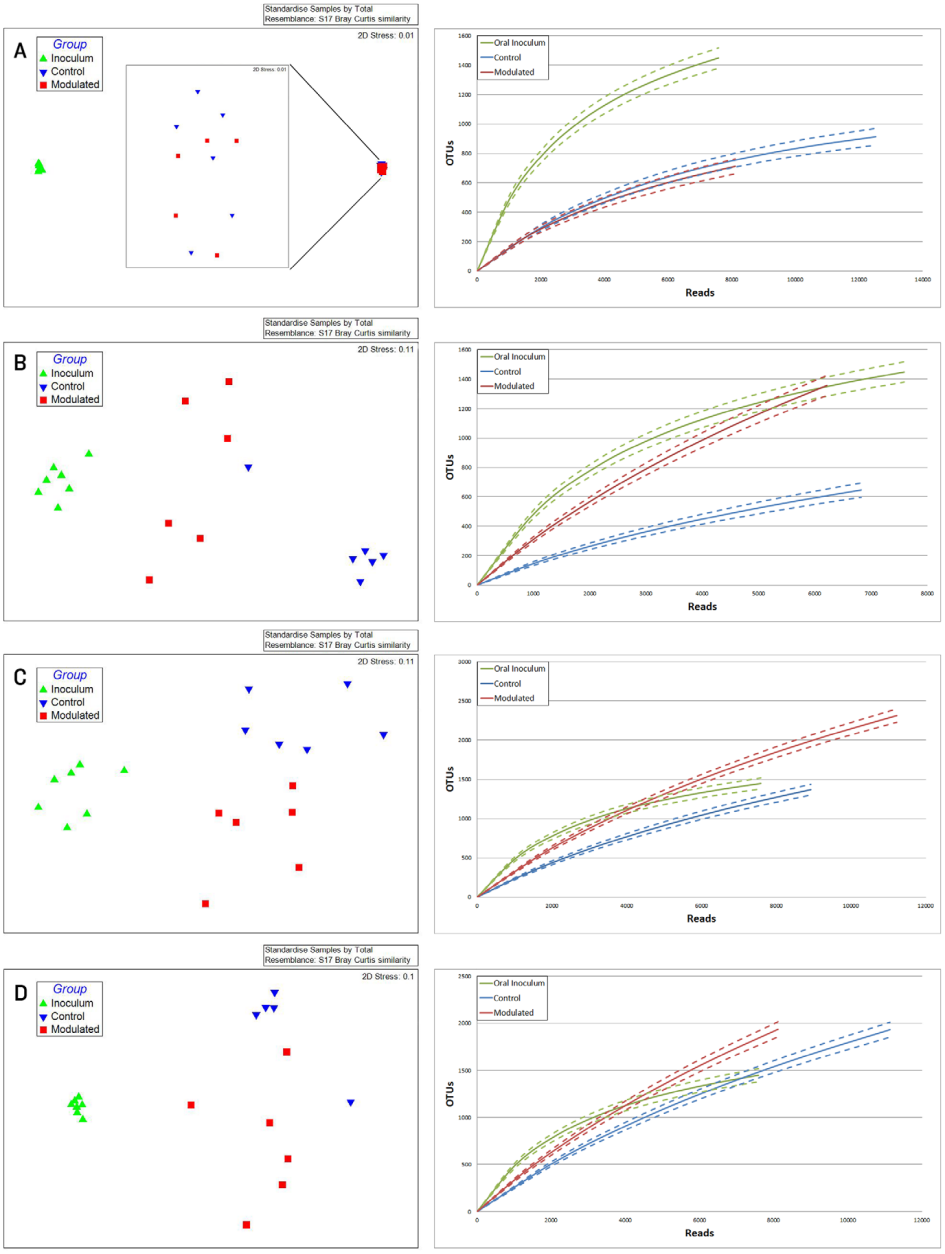
MDS plots and rarefaction curves of GI samples show successful modulation of GI microbial communities. MDS plots and rarefaction curves of GI microbiome samples A) 1 week before the start of oral inoculation (28 days of age; R = 0.056, *p* = 0.26), B) 1 day after completion of oral inoculation (40 days of age; R = 0.82, *p* = 0.002), C) at 56 days of age (R = 0.502, *p* = 0.002) and D) at 70 days of age (R = 0.483, *p* = 0.015).

**Figure 3:**
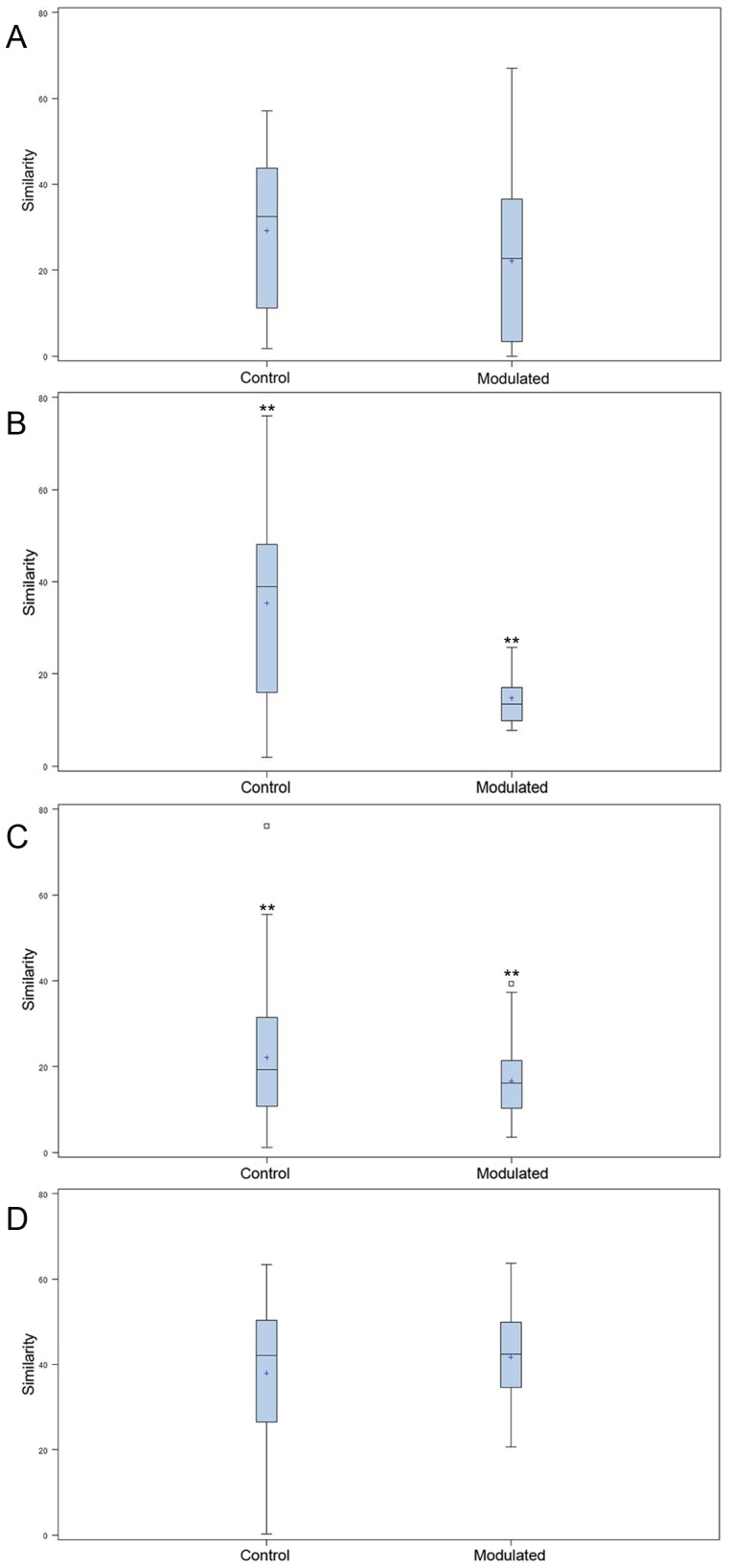
Differences in within group similarity for GI and respiratory microbiome samples at multiple time points. Boxplots showing no significant difference for within group similarity of the GI microbial communities A) for multiple time points before oral inoculation, B) significant differences for within group similarity (*p* = 0.0016) and variation (*p* = 0.0038) one day after completion of the oral inoculation (40 days of age) and C) significant differences for within group similarity (*p* = 0.0024) and variation (*p*<0.0001) for all time points following oral inoculation. D) Boxplots showing differences for within group similarity (*p* = 0.063) and variation (*p* = 0.0006) of the respiratory microbial communities for all time points following oral inoculation. ** denotes statistical significance of *p*<0.005.

Due to the immediate removal of the piglets from the gilt at birth, as well as the use of antibiotics during the first 4 weeks of life, only 0.70% of the OTUs in the modulated, and 0.55% of the OTUs in the control group GI samples at 27 days of age were found to be shared with the sow vaginal swab sample. 48.24% of OTUs were shared between the GI microbiomes of the modulated and control groups for all time points before GI modulation, and 42.43% for all time points after GI modulation. Successful modulation of the GI microbiome is evidenced by a significant increase (*p* = 0.023) in the number of OTUs present in the modulated group (445.83%) one day after oral inoculation (40 days of age) compared to the control group (20.98%). The modulated GI samples were found to share significantly more OTUs with the oral inoculum samples (13.06%) compared to the control GI samples (7.99%) after modulation (*p* = 0.0003). No difference in the number of OTUs shared with the oral inoculum samples was seen between the modulated (1.69%) and control group (1.36%) before modulation (*p* = 0.159). ANOSIM results and MDS plots of the GI microbiome samples revealed no significant difference in GI microbial composition between modulated and control groups before oral inoculation (ANOSIM R = 0.056, *p* = 0.26; Figure 2a). After 7 consecutive days of exposure to the oral inoculum, a statistically significant difference in the composition of the GI microbiomes was observed between the two groups (ANOSIM R = 0.82, *p* = 0.002; Figure 2b). This difference in GI microbiome composition was observed for the remainder of the study (Figure 2c & d). Analysis of the similarity between GI microbial samples within groups revealed no difference in similarity or variation before oral inoculation, and significantly less similarity and variation in the modulated group compared to the control group one day after oral inoculation (*p* = 0.0063 & *p* = 0.0038, respectively; Figure 3a & b). Significant differences in the within group similarity and variation in similarity were observed for all time points following oral inoculation (*p* = 0.0024 & *p*<0.0001, respectively; Figure 3c).

### Taxonomic Analysis of the Gastrointestinal Microbiome Samples

**Figure 4:**
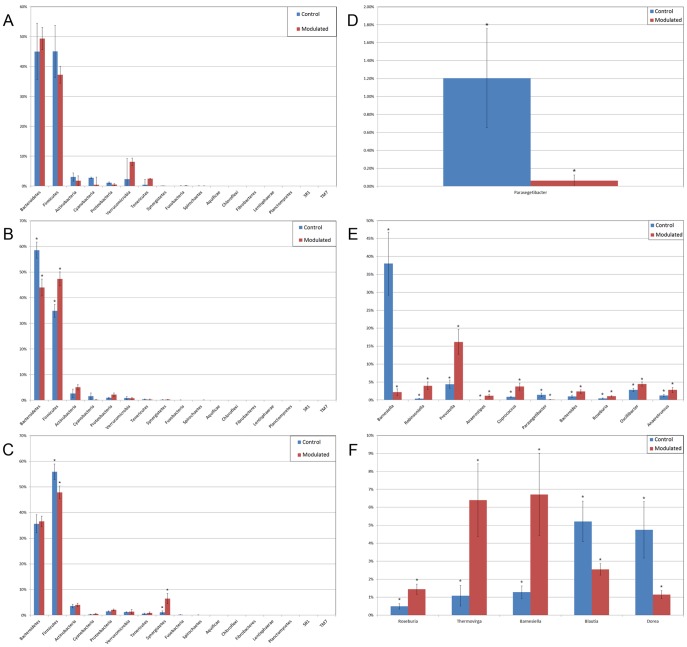
Differences in relative taxonomic abundances for GI microbiome samples at the phylum and genus level. The relative abundance of each phylum A) one day before oral inoculation (32 days of age), B) one day after completion of oral inoculation (40 days of age) and C) one day before euthanasia (103 days of age). The relative abundance of each genus showing statistically significant differences between the modulated and control group D) one day before oral inoculation (28 days of age), E) one day after completion of oral inoculation (40 days of age) and F) one day before euthanasia (103 days of age). * denotes statistical significance of *p*<0.05.

Results from the taxonomic analysis show no significant difference in the phylogenetic distribution of the two groups 1 day before oral inoculation (32 days of age), with Bacteroidetes (49.3% and 45%) and Firmicutes (37.2% and 45%) representing the dominant phyla in the modulated and control group, respectively (Figure 4a). One day after the completion of the oral inoculation (40 days of age), statistically significant differences in the relative abundance of both Bacteroidetes (44% and 58.5%, *p* = 0.0043) and Firmicutes (47.3% and 34.8%, *p* = 0.0036) phyla were visible between the modulated and control groups, respectively (Figure 4b). No significant differences were seen for any other phyla. A significant difference in the relative abundance of the Firmicutes phylum (47.9% and 55.9%, *p* = 0.0489) was still seen 1 day before euthanasia (103 days of age), as well as a significant difference in the relative abundance of Synergistetes (0.25% and 1.12%, *p* = 0.021) between the modulated and control groups, respectively (Figure 4c). A significant difference in the relative abundance of the Bacteroidetes phylum was no longer apparent at euthanasia, with a relative abundance of 36.6% and 35.6% in the modulated and control group, respectively.

Analysis at the genus level shows a significant difference in the relative abundance of only one genus (parasegetibacter, *p* = 0.026) 1 day before oral inoculation (32 days of age), with a relative abundance of 0.06% and 1.2% in the modulated and control group, respectively (Figure 4d). One day after oral inoculation (40 days of age) Barnesiella (2.17% and 38%, *p* = 0.0006), Prevotella (16.18% and 4.41%, *p* = 0.0035), Oscillibacter (4.45% and 2.79%, *p* = 0.0364), Robinsoniella (3.96% and 0.35%, *p* = 0.0026), Coprococcus (3.75% and 0.86%, *p* = 0.007), Anaerotruncus (2.8% and 1.2%, *p* = 0.0438), Bacteroides (2.36% and 0.99%, *p* = 0.0252), Anaerostipes (1.22% and 0.04%, *p* = 0.0064), Roseburia (1.03% and 0.42%, *p* = 0.0301) and Parasegetibacter (0.12% and 1.42%, *p* = 0.0091) were all found to have significantly different relative abundances in the modulated and control group, respectively (Figure 4e). Significant differences in the relative abundance of Barnesiella (6.7%% and 1.28%%, *p* = 0.02) and Roseburia (1.45% and 0.49%, *p* = 0.0043) were still seen 1 day before euthanasia (103 days of age), as well as significant difference in the relative abundance of Thermovirga (6.40% and 1.09%, *p* = 0.0123), Blautia (2.54% and 5.22%, *p* = 0.0225) and Dorea (1.15% and 4.75%, *p* = 0.0234) between the modulated and control groups, respectively (Figure 4f).

### OTU Analysis of the Respiratory Microbiome Samples

**Figure 5:**
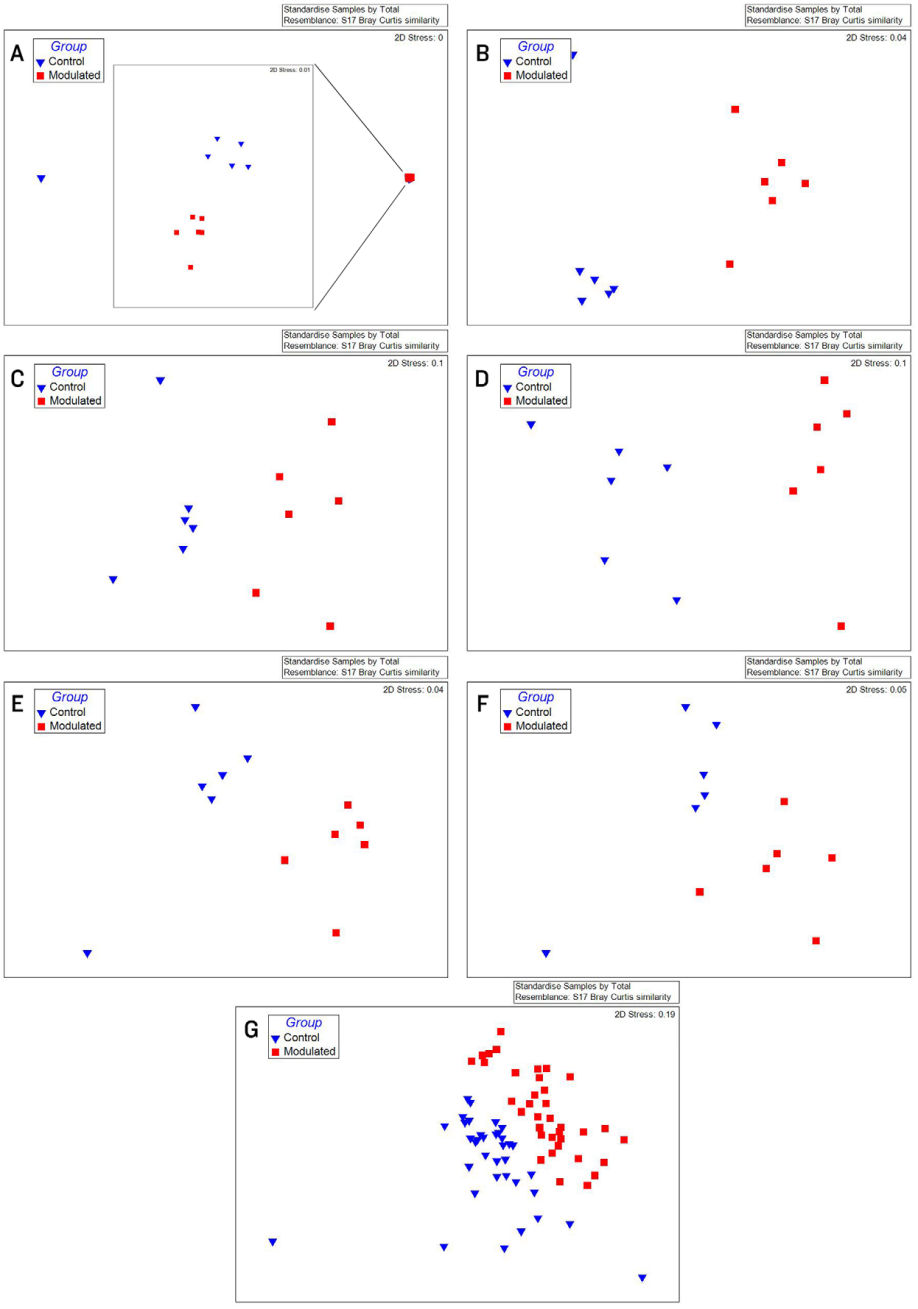
MDS plots for upper respiratory samples show successful modulation of respiratory microbial communities. MDS plots of upper respiratory microbiome samples A) the day of *M. hyopneumoniae* infection (69 days of age) (R = 0.667, *p* = 0.002), B) 7 days after *M. hyopneumoniae* infection (R = 0.763, *p* = 0.002), C) 9 days after *M. hyopneumoniae* infection (R = 0.704, *p* = 0.002), D) 12 days after *M. hyopneumoniae* infection (R = 0.807, *p* = 0.002), E) 14 days after *M. hyopneumoniae* infection (R = 0.719, *p* = 0.002) and F) 21 days after *M. hyopneumoniae* infection (R = 0.576, *p* = 0.002). G) MDS plot of all upper respiratory microbiome samples taken at multiple time points throughout the study (R = 0.368, p = 0.001).

0.70% of the OTUs in the modulated, and 0.56% of the OTUs in the control group upper respiratory samples taken throughout the study were found to be shared with the sow vaginal swab sample. 47.47% of OTUs were shared between the upper respiratory microbiomes of the modulated and control groups for all time points. 14.92% of OTUs were shared between the modulated upper respiratory and the oral inoculum samples, and 15.06% were shared between the control upper respiratory and oral inoculum samples, revealing no significant difference between the two groups (*p* = 0.8885). ANOSIM results and MDS plots of the upper respiratory microbiome samples revealed a statistically significant difference in the composition of the upper respiratory microbiomes between the two groups for all time points after oral inoculation (Figure 5). No respiratory microbiome samples were available for analysis prior to the oral inoculation. Lower respiratory tract samples showed no significant difference in microbial composition between the two groups (ANOSIM R = −0.029, *p* = 0.724; Data not shown). Analysis of the similarity between respiratory microbial samples within groups revealed the modulated group tended to be more similar (*p* = 0.063) and significantly less variable (*p* = 0.0006) compared to the control group for all time points following oral inoculation (Figure 3d).

### Taxonomic Analysis of the Respiratory Microbiome Samples

Results from the taxonomic analysis show Bacteroidetes and Firmicutes as the dominant phyla in both the modulated and control group’s upper respiratory microbiome samples for all available time points (69 to 91 days of age), with Bacteroidetes representing an average of 46.4% and 38.3% (*p* = 0.001) and Firmicutes representing an average of 44.9% and 51.8% (*p* = 0.002) in the modulated and control group after oral inoculation, respectively (Table 2). No respiratory samples were available before oral inoculation. Significant differences in the relative abundance of 4 phyla and 19 genera were seen between the two groups for all time points (Table 2). No significant differences in the relative abundance of any phyla or genera were detected between the two group in the lower respiratory microbiome samples (data not shown).

**Table 2:** Statistically significant differences in taxonomic abundance between the modulated and control group upper respiratory microbiome samples taken at multiple time points following oral inoculation.

Phylum	Control Upper Respiratory	Modulated Upper Respiratory	p-value
Actinobacteria	5.17%	3.91%	0.003
Bacteroidetes	38.30%	46.45%	0.001
Firmicutes	51.80%	44.88%	0.002
Synergistetes	0.14%	0.62%	0.0015
**Genus**	**Control Upper Respiratory**	**Modulated Upper Respiratory**	**p-value**
Janibacter	0.46%	0.11%	0.0071
Rothia	0.68%	0.14%	0.0005
Slackia	0.36%	0.08%	0.0211
Barnesiella	2.93%	1.83%	0.000999001
Tannerella	2.03%	3.49%	0.000999001
Paraprevotella	1.57%	2.74%	0.028
Rikenella	0.96%	2.74%	0.000999001
Pseudosphingobacterium	4.35%	6.35%	0.043
Aerococcus	0.50%	1.35%	0.000999001
Lactobacillus	0.41%	0.13%	0.0411
Streptococcus	2.65%	1.33%	0.000999001
Sarcina	0.05%	0.34%	0.0129
Mogibacterium	0.40%	0.11%	0.0307
Anaerotruncus	2.03%	1.15%	0.000999001
Sharpea	1.56%	2.11%	0.007
Solobacterium	0.90%	0.37%	0.0064
Sandaracinobacter	0.61%	0.27%	0.049598066
Succinivibrio	0.29%	0.70%	0.0284
Thermovirga	0.06%	0.51%	0.000395695

### Type I and IV Hypersensitivity

Allergic hypersensitivities are IgE mediated responses that typically occur within 20 to 30 minutes after re-exposure to a specific innocuous antigen. Type IV delayed type hypersensitivity (DTH) responses are cell-mediated reactions, which occur 2–3 days after antigen exposure. In this study a difference between the modulated and control group was observed for DTH responses to *Ascaris suum* (*A. suum*) skin testing following oral microbiota inoculation (54 days of age); however, no type I allergic responses were observed in the young pigs. The modulated group had a stronger DTH response than the control group for all allergen concentrations tested (data not shown). The largest DTH response difference between the two groups was observed at 100 µg *A. suum* extract, with the control group having an average increase in skin thickness of 0.643 mm, and the modulated group having an average increase of 1.19 mm (*p* = 0.07; Table 3).

**Table 3:** Summary of host responses.

Parameter Measured	Control	Modulated	P-Value
DTH (1,000 Units)	0.643	1.19	0.07
Cough Observations	9.6	4.4	<0.005
Lung Lesions (lobe area)	42%	28%	0.07
TNF-alpha Variance	5.8	0.18	0.0153

### Antibody Production

*M. hyopneumoniae* antibody levels in blood serum were monitored at 0, 2, 5, 7, 9, 12, 14 and 21 days post-infection (dpi). The results demonstrated seroconversion to *M. hyopneumoniae* in the modulated group prior to the control group. *M. hyopneumoniae* antibodies were detected in modulated animals as early as 9 dpi. At 12 dpi, five of the modulated pigs had seroconverted, compared to only two of the control animals. All pigs in both groups seroconverted by 14 dpi (Table 4).

**Table 4:** Seropositivity to *M. hyopneumoniae.*

Days Post Infection	Control	Modulated
0	0/6	0/6
2	0/6	0/6
5	0/6	0/6
7	0/6	0/6
9	0/6	1/6
12	2/6	5/6
14	6/6	6/6
21	6/6	6/6

Days post infection with *M. hyopneumoniae*. Seropositivity denotes number of animals with *M. hyopneumoniae* antibodies present in blood serum (6 animals/group).

### Respiratory TLR2 & TLR6 Transcription

Transcription levels of respiratory TLR2 (NM_213761.1) and TLR6 (NM_213760.1) were determined using frozen lung samples collected at euthanasia. Age-matched pigs (6) from a *M. hyopneumoniae* negative farm were used as a *M. hyopneumoniae* free baseline for TLR2 and TLR6 transcription levels. No significant difference was observed in respiratory TLR2 (*p* = 0.32) and TLR6 (*p* = 0.20) gene transcription levels between the modulated and control groups (Data not shown).

### Inflammatory Cytokine and C-reactive Protein Levels

**Figure 6:**
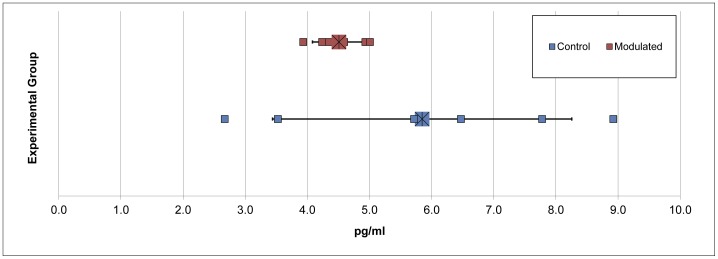
Differences in variation of BALF TNF-α levels. Results expressed as pg/ml of TNF-α in the BALF. Difference in the variance of the two groups is statistically significant (*p* = 0.0153).

Inflammatory cytokines IL-1β, IL-6, IL-8, TNF-α and C-reactive protein levels were monitored in the blood serum and bronchoalveolar lavage fluid (BALF). C-reactive protein serum levels were monitored throughout the study, and although an increase in C-reactive protein level was demonstrated following *M. hyopneumoniae* infection (data not shown), our results showed that there was no difference seen in these levels between the modulated and control groups. No statistical difference between the two groups was observed for cytokine levels in blood serum or BALF (data not shown). Despite no difference in the average TNF-α level of the two groups, a statistically significant difference (*p* = 0.0153) was observed for TNF-α variance in the BALF (Figure 6; Table 3). The modulated group demonstrated significantly less variation (0.18) than the control group (5.8) for BALF TNF-α levels.

### Bacterial Load

*M. hyopneumoniae* levels (CFU/ml) were determined using nasal swabs taken throughout the study, as well as bronchial swabs and BALF. CFU levels were not significantly different between the two groups, and the microscopic lung lesions suggestive of *M. hyopneumoniae* infection were apparent in each animal examined (*p* = 0.346; data not shown).

### Daily Weight Gain

Weights were recorded at 0, 15 and 22 days post infection (dpi) using a calibrated commercial animal scale. No significant differences in daily weight gain were observed between the modulated and control animals (data not shown).

### Coughing Scores

**Figure 7:**
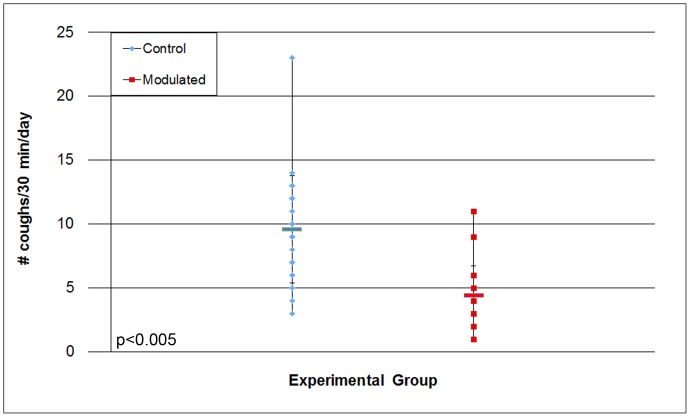
Differences in coughing scores of experimentally infected pigs. Scores were obtained by observing pigs for 30 min/d at the same time every day and recording the number of coughs per group. Observations started at 12 dpi and continued for the duration of the study (*p*<0.005).

Coughing levels for each group were recorded at the same time of day throughout the study. Coughing for the control group began as early as 7 dpi, whereas modulated animals did not begin coughing until 12 dpi, despite no difference in bacterial load between the groups. The number of dry coughs suggestive of *M. hyopneumoniae* infection was lower (*p*<0.005) in the modulated group than in the control group for the duration of the study (Figure 7). The average number of coughs/30 min was 9.57 in the control group and 4.39 in the modulated group (Table 3).

### Lung Lesions

**Figure 8:**
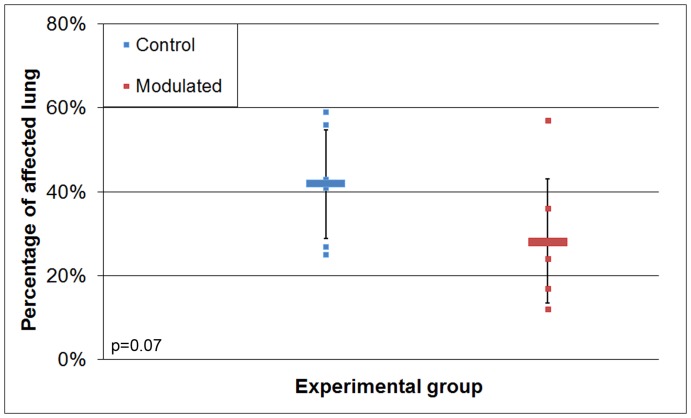
Differences in macroscopic lung lesion scores of pigs infected with *M. hyopneumoniae.* The lungs were removed at euthanasia (35 dpi) and evaluated blindly. Expressed as percentage of lungs with lesions (*p* = 0.07).

Lung lesion evaluations were done at euthanasia (35 dpi). Pigs in the modulated group tended to have lower (*p = *0.07) lung lesion scores, with an average of 28% of their lungs covered in lesions, compared to 42% in the control group (Figure 8; Table 3). Microscopic observations of lung lesions confirmed the presence of lesions caused by *M. hyopneumoniae* infection in all pigs. Perivascular and peribronchiolar lymphocyte infiltration, as well as increased numbers of mononuclear and polymorphonuclear cells in alveoli and lymphoid nodules were observed in all the pigs, each indicative of *M. hyopneumoniae* infection, as previously described [22]. No differences in microscopic lung lesions were observed between the two groups.

## Discussion

This study supports the hypothesis that modulation of GI microbiota can significantly affect systemic immune responses [23]. Results from this study clearly show modulation of the GI microbiome via oral inoculation, and a subsequent link between GI microbiota and systemic immune responses. This is evidenced by a stronger DTH response and less variation in respiratory TNF-α levels in the modulated group compared to the control group. The results from the coughing levels and lung lesion scores show a lower severity of infection in the modulated group than the control group as well, despite the lack of a difference in respiratory *M. hyopneumoniae* levels. All procedures performed in this study were applied to both the modulated and control group, with the exception of the oral inoculation. Because the oral inoculation was the only independent variable in the study, the results validate our hypothesis that modulation of GI microbiota has significant effects on systemic immune responses.

Increased incidence of allergies in developed countries is thought to be due to limited microbial exposure early in life [24]. In order to show the importance of early life microbial exposure on systemic immune response, a litter of newborn piglets was used as the experimental model, and modulation of the GI microbiota was performed at 33 days of age. Results from a previous study show delivery via caesarian results in infants being colonized by bacteria similar to those found on the skin surface [25]. Based on these results piglets in this study were born naturally as opposed to caesarean derived to ensure the piglet’s GI tract was colonized by microbes that would naturally colonize the neonatal gut, as opposed to skin derived microbes. Piglets were caught at birth using nitrile gloves and removed from the mother immediately to prevent contact with maternal feces and limit the vertical transfer of microbes. Analysis of the piglet microbiome samples and the gilt vaginal swab sample revealed that a minimal number of OTUs represented in the piglet microbiome samples originated from the sow. These results are not unexpected and likely due to the use of a medicated milk replacer, as well as antibiotic treatment during the first 4 weeks of the experiment. It is important to note that none of the piglets received maternal antibodies or immune molecules, as they were not allowed to suckle prior to being removed from their mother. The use of a single litter was designed to reduce genetic diversity (both individual and maternal) which could be responsible for differences seen in systemic immune responses between individuals. This is a potentially important aspect of the study, as both individual genetic and maternal factors can have an effect on immune function. Adding a second litter with a different genetic background (both individual and maternal) introduces another variable into the experiment that could have significant effects on immune responses.

Taxonomic abundances of the modulated and control group 1 day before oral inoculation (32 days of age) were comparable to normal pigs, with Firmicutes and Bacteroidetes representing the dominant phyla. Only the relative abundance of the low abundance parasegetibacter genus was significantly different between the two groups before oral inoculation. Significant differences in the relative abundance of 2 phyla and 10 genera in the GI microbiome samples of the two groups were observed one day after oral inoculation. Chao1 estimates, Shannon’s diversity index and the number of OTUs present in the GI microbiome samples were all increased in the modulated group compared to the control group after oral inoculation. MDS and ANOSIM analysis shows the GI microbial communities are more similar within groups than between following oral inoculation. These results, in addition to the increase in the number of OTUs shared between the modulated group GI microbiome and the oral inoculum samples compared to the control group confirm the successful modulation of the GI microbial community.

Despite no difference in richness or diversity between the upper respiratory samples of the two groups, significant differences in the relative abundance of 4 phyla and 19 genera were observed for all time points following oral inoculation, with Bacteroidetes and Firmicutes representing the dominant phyla in both groups. MDS and ANOSIM analysis shows the upper respiratory microbial communities are more similar within groups than between following oral inoculation. No samples were available for analysis prior to oral inoculation. These results in addition to similar numbers of OTUs shared between the upper respiratory and oral inoculum samples of the two groups suggest an indirect modulation of the upper respiratory microbial community via direct modulation of the GI microbial community.

The GI microbiota are constantly sampled by the host and participate in stimulation of the immune system [26]. This stimulation is believed to be important in establishing baseline immune responses to pathogenic infection. Previous studies have shown that a lack of GI microbial diversity is associated with increases in allergic disease, as well as reduced immune responses [27], [28], [29]. Both allergic and delayed type hypersensitivity tests were conducted to test for differences in allergic sensitivity and baseline systemic immune responses. No allergic responses were observed for any of the pigs in this study at the time of testing (54 days of age). The lack of an allergic response to the *A.* suum antigen is proposed to be due to the young age of the pigs at the time of exposure. It is possible that the pigs were not given sufficient time to develop an allergy to the *A. suum* antigen, and that testing at a later date during the experiment may be ideal for future studies. This theory is derived from the fact that this antigen has been used in the past with older pigs, yielding positive allergic responses [30]. Despite a lack of IgE meditated type I allergic response to the *A. suum* skin testing, the DTH results established stronger systemic immune responses for the modulated group caused by the modulation of the GI microbiota.

The disease progression and severity of *M. hyopneumoniae* infection can rely on a number of factors, from the immune status of the pig, to the *M. hyopneumoniae* bacterial load, to co-infection with other respiratory pathogens [23]. This study was conducted in a controlled research facility and pigs were tested for porcine respiratory and reproductive syndrome virus (PRRSv) and swine influenza virus (SIV) antibodies in order to show a lack of co-infection. To show the differences seen in the severity of infection were due to differences in systemic immune responses caused by GI microbial modulation, and not differences in disease progression, bacterial load was determined from nasal swabs taken throughout the study, as well as bronchial swabs and BALF. These results showed no difference in bacterial load between the two groups for any time point throughout the study.

Results from the coughing observations show a significant decrease in the number of coughs/30 minutes, and lung lesions were found to cover a smaller percentage of the lungs in the modulated group compared to the control group. Although the source of an individual cough could not be assigned to an individual pig, the location of the coughs within the pen suggests that all of the pigs contributed to the total coughing score. Furthermore, the subsequent lung lesion data is consistent with a distribution among the group rather than a single pig being responsible for the increased group average.

An important aspect of this study is the idea that the GI microbiota are involved in the modulation of the systemic immune system, having a direct effect on systemic immune responses, and that responses are not due to any interaction between GI microbiota and the pathogens used as systemic immune response triggers. In order to show this effect it was important to keep the GI microbiota and pathogenic challenges contained from one another. The lack of direct contact between GI microbiota and the pathogenic challenges requires there to be GI microbial regulation of the systemic immune system to explain the differences in systemic immune responses. *M. hyopneumoniae’s* restriction to the respiratory tract [31] fulfils these requirements. This intestinal-free stimulant has no direct contact with the GI microbiota, and therefore shows that systemic immune responses were altered by GI microbiota in the modulated group. Similar numbers of shared OTUs between the oral inoculum and upper respiratory microbiome samples of both groups (*p* = 0.8885) show that the GI modulation did not directly cause modulation in the respiratory tract, and these differences were therefore an indirect result of the altered GI microbial communities.

There is no evidence that we are aware of to suggest that differences in the composition of the respiratory microbiome have effects on *M. hyopneumoniae* infections. The pigs were tested for and found to be free of other prominent respiratory infections (SIV and PRRSv), showing that co-infection was not a factor in this study. The lack of a difference in *M. hyopneumoniae* bacterial load throughout the study also indicates that there was no modulation of the *M. hyopneumoniae* infection (the disease progression was the same, but immune responses were different). Furthermore, the differences in DTH response, antibody production, and TNFα variance observed in this study are all examples of altered systemic immune responses due to microbial modulation.

The acute phase response is an early non-specific immune response to infection and involves the induction of serum proteins known as acute phase proteins [32]. C-reactive protein is an acute phase protein that binds to dying cells, as well as bacteria to activate the complement system [33]. It has been shown that C-reactive protein levels increase in pigs infected with *M. hyopneumoniae* [34]. In addition to C-reactive protein, proinflammatory cytokines are known to play important roles in porcine immune response to infection. IL-1β is known to induce IL-6 production, and IL-8 has been reported to be a chemotactic for T cells and neutrophils [35]. TNF-α is also an important factor in porcine disease, being responsible for the accumulation of lymphocytes in *M. hyopneumoniae* infections [36]. Increases in all four of these cytokine levels have been reported in pigs infected with *M. hyopneumoniae* [35], [37]. Because of their importance in systemic immune responses, as well as their association with *M. hyopneumoniae* infection, these immune response elements were monitored throughout the study. Although increased levels were observed as the study went on, no significant differences between the two groups were seen, with the exception of the level of variation seen in the TNF-α levels of the BALF. The modulated group had significantly less variation in their TNF-α levels compared to the control group, suggesting a tighter regulation of TNF-α levels in the lungs due to GI modulation.

It was thought that the reduced variation in the modulated group TNF-α levels could be due to higher within group similarity in the GI samples of the modulated group. Surprisingly, within group similarity was significantly lower in the modulated group than the control group following oral inoculation. Despite the lowered similarity, the variation in the similarity within the modulated group was significantly reduced following oral inoculation. This significantly lower variation in within group similarity was also seen in the respiratory samples of the modulated group. The reduced variation of within group similarity in the modulated group correlates with the reduced variation in TNF-α levels in the BALF, however it is unclear what mechanisms are responsible for this regulation.

*M. hyopneumoniae* has been revealed as a ligand for porcine TLR2 and TLR6, and the stimulation of porcine alveolar macrophages with *M. hyopneumoniae* has been shown to induce TNF-α production in vitro [36]. Furthermore, these studies showed that TNF-α production could be blocked using antiporcine TLR2 and TLR6 antibodies. Because of the connection between TLR2, TLR6 and TNF-α production it was our theory that the variation in TNF-α levels seen in the BALF could be accounted for by differences in TLR expression levels caused by the oral inoculation. In order to test this theory, respiratory transcription levels of TLR2 and TLR6 were analysed for the control and modulated groups of infected pigs as well as a third group (age matched pigs which had not been infected with *M. hyopneumoniae*). The results of these studies showed no significant difference in the expression levels or variances of TLR2 and TLR6 in the lungs of any group studied. However the much tighter regulation of the subsequent TNF-α production associated with the modulated group suggests that tighter regulation of immune response genes in the lungs is associated with GI microbial diversity, and further studies are required to determine what mechanisms are responsible for this regulation.

From these results we conclude that a non-pathogenic oral inoculum successfully modulated the GI microbial community, significantly regulated the systemic immune system of the pig and lowered the severity of infection. This hypothesis is supported by the stronger DTH response, the decreased severity of infection, and the significantly lower amount of variation seen in TNF-α levels in the lungs of the modulated group. Further studies need to be done to determine how the GI microbiota regulates immune responses outside the gut.

## Materials and Methods

### Ethics Statement

This study was approved by the Institutional Animal Care and Use Committee (IACUC) and the Institutional Biosafety Committee of the University of Illinois at Urbana-Champaign (protocol # 09141 and 09146). All animals were cared for following the guidelines of the IACUC and the Institutional Biosafety Committee of the University of Illinois at Urbana-Champaign, and all efforts were made to minimize suffering throughout the study.

### Experimental Design

A litter of pigs (12) was removed from their mother immediately following birth in order to prevent exposure to the maternal GI microbiota. The pigs were raised in controlled research units and fed medicated milk replacer until weaning (28 days old). At 33 days of age the pigs were randomly assigned to 2 groups based on weight and gender, one of which was inoculated (modulated) with the GI microbiota from a healthy adult boar for seven consecutive days, while the other was not (control). Nasal swabs and fecal samples were collected throughout the study and sequenced to determine the effects of the oral inoculation on GI and respiratory microbial communities. In order to determine the effectiveness of the GI microbial modulation on porcine systemic immune responses, allergic and delayed type hypersensitivity responses (type I and IV, respectively) were measured in both groups via *A. suum* worm extract skin testing at 54 days of age, and pigs were experimentally infected with *M. hyopneumoniae* at 69 days of age and observed for 5 weeks. Various systemic immune responses and severities of infection were analyzed throughout the study including: *M. hyopneumoniae* antibody production, respiratory TLR2 & TLR6 transcription levels, and cytokine and C-reactive protein levels.

### Source of Animals and Housing

A pregnant gilt obtained from a high health herd was housed under commercial conditions and then transferred to a research facility 3 weeks before farrowing. The gilt was induced with 3 doses of 10 mg dinoprost tromethamine (Lutalyse®, Pfizer Inc., New York, NY, USA) intramuscularly at 12 hour intervals starting at day 113 of gestation. Plastic was placed under the gilt and piglets were caught at birth using nitrile gloves to prevent contact with fecal matter. The piglets were removed immediately following birth and raised in controlled research units in order to prevent exposure to GI colonizing microbiota. Research suites were equipped with HEPA filters and the ventilation system was individualized for each room. Biosecurity measures were followed at all times to avoid cross-contamination between experimental groups.

### Feeding Protocol

To avoid vertical transfer of porcine immune molecules, piglets were removed from gilts before suckling and syringe fed 20–25 mL of previously frozen bovine colostrum, obtained from the University of Illinois Dairy Farm, every 2 hours for the first 48 hours of life. The colostrum tested negative for *M.* hyopneumoniae antibodies. Piglets were then switched to Advance Liqui-Wean Medicated Pig Milk Replacer® (Oxytetracycline and Neo-Terramycin) (MSC, Carpentersville, IL, USA), which was pumped into bowls every 60 min at a rate of 360 mL/kg/day. Both antibiotic and colostrum were used in order to ensure the piglets health, as previous attempts by our group to artificially raise piglets without colostrum or antibiotic treament resulted in severe *E. coli* infections and gastrointestinal clinical signs. In order to prevent respiratory infection and gastrointestinal clinical signs, Baytril (enrofloxacin) was injected subcutaneously into the ear in 100mg/30lbs body weight doses 24 hours after birth. To prevent gastrointestinal infection, neomycin sulfate was administered orally at a rate of 10 mg/lb body weight every day for the first 2 weeks of life. At 10 days of age the piglets were introduced to phase I dry feed and were eventually weaned off the milk replacer over a 2-day period once they reached an average weaning weight of 6 kg (28 days of age). Piglets were kept on the dry feed *ad libitum* for the remainder of the study.

### Collection, Preparation and Delivery of Oral Inoculum

Fresh feces was collected daily from a single boar from a high health herd (*M. hyopneumoniae,* PRRSv, *Pasteurella multocida* and *Bordetella bronchiseptica* free) for use as an oral inoculum. The farm has clinical and historical data backing up their high health status. Depopulation and repopulation had occurred just months before the samples were taken. The boar was showing no clinical signs of infection at the time of collection, and flotation tests done by the University of Illinois Veterinary Diagnostic lab were negative for GI parasites. Samples were immediately mixed 1∶1 with sterile phosphate buffered saline (PBS) and fed by syringe to the 33 day old piglets (modulated) at a rate of 2 mL/Kg, as previously described [38]. This process was repeated for 7 consecutive days in order to ensure GI colonization.

### *Ascaris suum* Antigen Hypersensitivity Testing

Starting at 33 days of age, pigs in both groups were sensitized to *A. suum* antigen by two bi-weekly injections containing 1 mg of *A. suum* worm extract mixed in alum. Solution was made by adding 0.05 µl aluminum potassium sulfate and 24 µl of sodium bicarbonate for every mg of *A. suum* worm extract. The solution was then mixed and allowed to stand at room temperature for 30 min, and then overnight at 4°C. The solution was centrifuged, the supernatant was removed, and the precipitate was resuspended in distilled water at the desired concentration (worm extract and protocol kindly provided by F. Zuckermann, College of Veterinary Medicine, UIUC). Injections (SC) were given in the abdominal wall. One week after the final injection (54 days of age), skin tests for hypersensitivity type I and IV were performed. Pigs were anesthetized in order to obtain accurate measures of antigenic responses. A mixture of 1.5 mg/kg xylazine and 8 mg/kg of a commercial formulation of tiletamine and zolazepam (Telazol®, Fort Dodge Animal Health, Fort Dodge, IA, USA) was used for anesthesia. The skin test consisted of 10 intradermal injections in the abdominal wall performed in duplicate for each pig. The injections consisted of 100 µl of four-fold serially diluted *A. suum* worm extract (4,000; 1,000; 250; 62.5; 15.6; 3.9; 0.97; 0.24; 0.06; 0.015 µg/ml). Saline (100 µl) was also injected as a negative control. Type I hypersensitivity was measured 20 minutes after *A. suum* worm extract injection. Pigs were anesthetized again 24 hours after skin testing to measure for type IV hypersensitivity. Calipers were used to determine skin thickness of injection sites in mm, as previously described [21].

### Experimental Infection with *M. hyopneumoniae*

69 day old pigs were experimentally inoculated with 10 ml of a 2x10^5^ color changing units (ccu/ml) lung homogenate containing *M. hyopneumoniae* strain 232 (purchased from Iowa State University, Ames, IA, USA) using intra-tracheal intubation to guarantee uniform infection throughout the groups. Due to the fact that growing *M. hyopneumoniae* colonies on agar plates is difficult and requires weeks of incubation, ccu/ml is the standard technique used for determine the concentration of *M. hyopneumoniae* [39]. The ccu/ml technique uses 10-fold serial dilutions of mycoplasma in Friis broth containing a pH sensitive color indicator, usually phynol red, which changes color depending on the acidity of the media. The color changes from red to yellow due to acidification by the cell’s metabolism during growth. This change in color is used to determine the concentration of mycoplasma. Endotracheal tubes, syringes and needles employed for inoculation and injection were sterile (individually wrapped) and a different set was used for each animal. The pigs were anesthetized using the same protocol as for the *A. suum* sensitization. An endotracheal tube was placed in the trachea using the lighted guide of a laryngoscope, and the *M. hyopneumoniae* inoculum was administered to animals through the endotracheal tube, as previously described [40].

### Observation of Clinical Signs

Starting at 12 dpi the control and experimental groups were observed 30 minutes/day for coughing rates. While remaining out of sight, observations were scored for each group by listening for coughing, as previously described [41], [42]. Coughing scores were recorded by group and not individual pigs in order to minimize stress levels during observation which could lead to inaccurate coughing scores. Observations were performed at the same time each day.

### Weight Measure

Pigs were individually weighed at the same time of day at 0, 15 and 22 dpi using a calibrated commercial animal scale.

### Sample Collection

#### Sow Vaginal Swab

Sow vaginal swab was collected two days before birth by introducing a sterile BD CultureSwab® (Becton Dickinson and companies, Franklin Lakes, NJ, USA) into the vagina of the gilt and rotating it clockwise and counter clockwise, and stored at −80°C.

#### Blood Serum

Blood samples were obtained from all pigs at the same time of day at 0, 2, 5, 7, 9, 12, 14, and 21 dpi. Samples were collected in BD Serum Vacutainers® (Becton Dickinson and Company, Franklin Lakes, NJ, USA) and centrifuged at a rate of 3,000 rpm for 10 minutes. Serum was then removed from the tubes and stored at −80°C in sterile 1.5 mL Eppendorf tubes. ELISA for *M. hyopneumoniae,* PRRSv and SIV antibodies were performed in order to determine the rate of antibody production, as well as rule out the possibility of other respiratory diseases. Observation of C reactive protein, interleukins IL-1β, IL-6, IL-8, and TNF-α blood levels were performed by ELISA, as described in the determination of C-reactive protein, IL-1β, IL-6, IL-8, TNF-α, and specific *M. hyopneumoniae* antibodies section.

#### Fecal Samples

Fecal samples were collected daily from the piglets starting one week after birth and continuing up until euthanasia. Samples were collected in Whirl-pak sample bags (Nasco, Fort Atkinson, Wisconsin, USA) individually for each pig and stored at −20°C.

#### Nasal and Bronchial Swabs

Nasal swabs were collected at 0, 7, 9, 12, 14, and 21 dpi by introducing approximately 4 mm of a sterile BD CultureSwab® (Becton Dickinson and companies, Franklin Lakes, NJ, USA) into each pig nostril and rotating it clockwise and counter clockwise. Bronchial swabs were collected at euthanasia using the same type of sterile swabs used for nasal swabbing and rotating them in the bronchia instead of the nostrils. All swabs were stored at −80°C.

#### Lung Tissue

Both healthy lung and portions containing lesions were collected at euthanasia. Samples were taken from the same location on the right antero-ventral lobe and placed in sterile 50 mL conical tubes. These tubes were flash frozen in liquid nitrogen and placed at −80°C. Frozen lung samples were used in the gene expression level experiments of porcine TLR2 and TLR6. Samples of both healthy lung and portions containing lesions were stored in 10% fixative as well. Fixed samples were stained with H&E and used in microscopic evaluation of lung lesions.

#### BALF

Bronchoalveolar lavage fluids (BALF) were collected at euthanasia. Cytokine levels associated with *M. hyopneumoniae* infection were analyzed in the BALF. Sterile PBS (20 ml) was introduced into the bronchoalveolar space, massaged through the lungs and re-collected in 50 mL conical tubes. BALF was stored at −80°C.

### Lung Lesion Evaluation (Macroscopic and Microscopic)

Macroscopic lung lesion evaluations were done as a single blind study following euthanasia. Lung lesions were scored based on the percentage of lung covered in lesions suggestive of *M. hyopneumoniae* infection, as previously described [43]. Macroscopic lung lesions indicative of *M. hyopneumoniae* infection are purple or grey with a rubbery consolidation, have increased firmness, a failure to collapse and are marked by edema of the lungs [44]. Microscopic lung lesion evaluation was done using the fixed lung tissues collected at euthanasia. Samples were embedded in paraffin, sectioned onto slides for evaluation and observed through an optic microscope [45].

### Determination of Bacterial Load in Nasal and Bronchial Swabs

DNA from nasal swabs, bronchial swabs, and BALF were extracted using the DNeasy Blood and Tissue Kit (Qiagen, Valencia, CA, USA). Extracted DNA was submitted to the Veterinary Diagnostic Laboratory at the University of Minnesota (VDL-UMN) for quantification of the bacterial load by Real Time PCR VetMAX™ (Life Technologies Corporation, Carlsbad, CA, USA) with *M. hyopneumoniae* specific reagents and controls. DNA was also stored at −20°C for use in microbiome sequencing analysis.

### Determination of C-reactive Protein, IL-1β, IL-6, IL-8, TNF-α, and Specific *M. hyopneumoniae* Antibodies

*M. hyopneumoniae* antibodies, cytokine and C-reactive protein levels were measured in blood serum and BALF. Pig serum samples were submitted to the VDL-UMN for determination of *M. hyopneumoniae* antibodies using the DAKO® ELISA test [46]. Cytokines were measured in the serum and BALF using a porcine specific multiplex ELISA test (Aushon Searchlight, Aushon Biosystems Inc., Billerica, MA, USA). C-reactive protein in serum was measured using the PHASE® (Tridelta Development Ltd, Maynooth, Ireland) ELISA assay kit following the manufacturer’s instructions.

### RNA Isolation, cDNA Synthesis, and TLR2 and TLR6 Gene Expression Analysis

Total RNA was isolated from snap frozen lung tissue of all animals experimentally infected with *M. hyopneumoniae,* as well as 6 age-matched pigs from a *M. hyopneumoniae* negative farm. The latter group of pigs was used as a control for the gene expression level analysis. The RNeasy Mini Kit (Qiagen, Valencia, CA, USA) was used for RNA extraction from homogenized tissue following the manufacturer’s instructions. Total mRNA samples were treated with DNase I (Qiagen, Valencia, CA, USA) in order to remove genomic DNA contamination, and concentrations were determined using an Eppendorf Biophotometer (Eppendorf, Westbury, NY, USA). Two µg of total RNA was used for reverse transcription using the Omniscript RT Kit (Qiagen, Valencia, CA, USA) in a 20µl reaction containing 10 µM Oligo-(dT), 10 U RNase inhibitor, 5 mM of each dNTP, RT buffer and 1 U of Omniscript RT for each sample. The reaction was allowed to occur at 37°C for 90 min. Negative controls, which contained no reverse transcriptase, were processed identically with the samples. Quantitative expression of TLR2, TLR6 and 18s genes was investigated using real-time PCR. The 18s gene was used as an internal control. Gene-specific primers for TLR2 were designed with Primer Express® software (Applied Biosystems, Foster City, CA, USA) and the sequences were: forward primer (5′-GGGCTCTGTGCCACCACTT-3′) and reverse primer (5-GGAGCCAGGCCCACAATC-3′). Gene-specific primers (18s) were obtained from previous publications [47]. Quantitative expression of TLR2 and 18s was performed using SYBR® Green PCR Master Mix, while TLR6 expression was determined using a specific TaqMan® Gene Ex Assay (assay ID Ss03392239_s1), both of which were obtained from Applied Biosystems (Foster City, CA, USA). Ten-fold serial dilutions of each gene were prepared from cDNA and a non-template control, and used for the standard curves to determine PCR efficiencies. Real-time PCR was performed in an ABI 7900HT fast real-time PCR system (Applied Biosystems, Foster City, CA, USA). The PCR amplification program steps were 10 min at 95°C , then 40 cycles of: 15 s at 95°C, 1 min at 60°C, 1 min at 72°C, and a final cycle for dissociation analysis of 15 s at 95°C and 15s at 60°C. All samples were run in triplicate, including all dilutions in the standard curve. Automatic cycle threshold (Ct) values obtained from the real-time PCR for TLR2 and TLR6 were normalized using the 18s rRNA transcript. PCR amplification efficiencies and correlation coefficients were analyzed. Final data for relative quantification of gene expression was obtained by applying the comparative Ct method (ΔΔCt) [48].

### Microbiome Analysis

#### DNA Extraction

DNA from fecal and sow vaginal samples were extracted using the QIAamp DNA Stool Mini Kit (Qiagen, Valencia, CA, USA), and DNA from nasal swabs, bronchial swabs, and BALF were extracted using the DNeasy Blood and Tissue Kit (Qiagen, Valencia, CA, USA).

#### Sequencing

DNA extracted from nasal swabs, lung lavage, bronchial swabs, vaginal swabs and fecal samples was subject to 454 pyrosequencing of the V1-V3 region of the 16S rRNA gene. PCR primers flanking the V1-V3 hypervariable region of the bacterial 16S rRNA gene were designed for amplification. The oligonucleotide primers were HPLC-purified and included an A or B sequencing adapter at the 5′ end and template specific sequences at the 3′ end. Barcodes were located between the A sequencing adapter and the template specific sequence of the forward primer. The primer sequences were: 5′ CCATCTCATCCCTGCGTGTCTCCGACTCAG – BARCODE – AGAGTTTGATCCTGGCTCAG 3′ (forward) and 5′ CCTATCCCCTGTGTGCCTTGGCAGTCTCAG – ATTACCGCGGCTGCTGG 3′ (reverse). The PCR amplification mixture contained 1.25 units HotStarTaq Plus DNA Polymerase (Qiagen, Valencia, CA, USA), 2.5 µl 10X PCR Buffer (Qiagen, Valencia, CA, USA), 2 mM dNTPs, 10 mM forward and reverse primer and 5–20 ng DNA in a reaction volume of 25 µl. The PCR conditions were an initial denaturation at 95°C for 15 minutes, followed by 35 cycles of 95°C for 15 seconds, 65°C for 45 seconds, 72°C for 1 minute, and a final 10 minute elongation at 72°C. Samples were run on a 1.5% agarose gel to verify product amplification. The PCR products were cleaned up using the Agencourt AMPure XP beads kit (Beckman Coulter, Inc., Brea, CA, USA). The PCR products were pooled into groups of 15 in equal concentration ratios based on the quantification results using the NanoDrop 1000. The pooled PCR amplicons were sequenced using 454 FLX-Titanium technology at the W.M. Keck Center for Comparative and Functional Genomics (University of Illinois, Urbana, IL).

### Data Analysis

Data was evaluated using the Students *t* test or Kruskall-Wallis one-way analysis of variance, were appropriate. Equality of variances was measured using the Brown-Forsythe homogeneity of variance test. The proportion of seropositve pigs was compared using a Hypothesis test. The pig was the experimental unit for all comparisons. Following sequencing, 16S rRNA gene reads were assessed for quality. Sequences shorter than 200 nucleotides, with homopolymers longer than 6 nucleotides, containing ambiguous base calls, or with an average quality score <30 were removed. Sequences were aligned against the silva database [49]. Potentially chimeric sequences were detected using mothur’s [50] implementation of UCHIME [51] and removed. The remaining reads were pre-clustered as previously described [52] and then clustered using ModalClust (https://bitbucket.org/msipos/modalclust). OTUs were defined as sharing ≥97% sequence complete-linkage identity with the most abundant sequence forming the OTU seed. OTUs detected in less than three samples and fewer than three times were removed as possible artifacts. The relationships among the samples was compared using Bray-Curtis dissimilarity statistics following normalization of the data to their total read depth (i.e. the proportional representation of each OTU) and transformation of this data by square root to reduce the influence of higher abundant over less abundant OTUs. The total number of bacteria in each sample was not reported, as it is our position that relative abundance is far more informative. This view is supported by previous microbiome studies in which total number of bacteria are not reported [7], [8], [9], [10], [53], [54], [55], [56], [57], [58]. Shannon’s diversity indices were performed in R using the Vegan package [59]. Bias-corrected Chao1 richness estimates were obtained in mothur [50] using methods described previously [60]. Resemblance matrices and non-metric multidimensional scaling (MDS) plots were constructed using this data and visualized in Primer6 [61]. Boxplots were constructed and analysis of the resemblances was done using SAS software, Version 9 of the SAS System for Windows. Copyright © 2011 SAS Institute Inc. SAS and all other SAS Institute INc. product or service names are registered trademarks or trademarks of SAS Institute Inc., Cary, NC, USA. Taxonomic profiles were generated for all reads using the RDPclassifier v2.4 [62] with a cutoff of 0.7. Detection of differentially abundant taxonomic groups was done using Metastats [63].
